# Impact of *MMP2* rs243865 and *MMP3* rs3025058 Polymorphisms on Clinical Findings in Alzheimer's Disease Patients

**DOI:** 10.1155/2021/5573642

**Published:** 2021-04-19

**Authors:** Vladimira Durmanova, Juraj Javor, Zuzana Parnicka, Gabriel Minarik, Agata Ocenasova, Barbora Vaseckova, Veronika Reznakova, Maria Kralova, Tomas Hromadka, Ivana Shawkatova

**Affiliations:** ^1^Institute of Immunology, Faculty of Medicine, Comenius University in Bratislava, Bratislava, Slovakia; ^2^Department of Molecular Biology, Faculty of Natural Sciences, Comenius University in Bratislava, Bratislava, Slovakia; ^3^Psychiatry Outpatient Clinic, University Hospital and Polyclinic the Brothers of Saint John of God in Bratislava, Slovakia; ^4^Care Centre Centrum Memory, Bratislava, Slovakia; ^5^Clinic of Psychiatry, Faculty of Medicine, Comenius University in Bratislava and University Hospital, Bratislava, Slovakia; ^6^Institute of Neuroimmunology, Slovak Academy of Sciences, Bratislava, Slovakia

## Abstract

Alzheimer's disease (AD) is a chronic neurodegenerative disease of the central nervous system with higher prevalence in elderly people. Despite numerous research studies, the etiopathogenesis of AD remains unclear. Matrix metalloproteinases (MMPs) are endopeptidases involved in the cleavage of extracellular matrix proteins and basement membrane compounds. In the brain, the pathological role of MMPs includes the disruption of the blood-brain barrier leading to the induction of neuroinflammation. Among various MMPs, MMP-2 and MMP-3 belong to candidate molecules related to AD pathology. In our study, we aimed to evaluate the association of *MMP2* rs243865 and *MMP3* rs3025058 polymorphisms with AD susceptibility and their influence on age at onset and MoCA score in patients from Slovakia. Both MMP gene promoter polymorphisms were genotyped in 171 AD patients and 308 controls by the PCR-RFLP method. No statistically significant differences in the distribution of *MMP2* rs243865 (-1306 C>T) and *MMP3* rs3025058 (-1171 5A>6A) alleles/genotypes were found between AD patients and the control group. However, correlation with clinical findings revealed later age at disease onset in *MMP2* rs243865 CC carriers in the dominant model as compared to T allele carriers (CC vs. CT+TT: 78.44 ± 6.28 vs. 76.36 ± 6.39, *p* = 0.036). The results of *MMP3* rs3025058 analysis revealed that 5A/6A carriers in the overdominant model tended to have earlier age at disease onset as compared to other *MMP3* genotype carriers (5A/6A vs. 5A/5A+6A/6A: 76.61 ± 5.88 vs. 78.57 ± 6.79, *p* = 0.045). In conclusion, our results suggest that *MMP2* rs243865 and *MMP3* rs3025058 promoter polymorphisms may have influence on age at onset in AD patients.

## 1. Introduction

Alzheimer's disease (AD) is a chronic neurodegenerative disease of the central nervous system characterized by progressive memory loss, confusion, and cognitive dysfunction. It is the cause of 60 to 70% of dementia cases. The AD prevalence is estimated at 4.4% in people aged 65 years to 22% in people aged 90 years and older [1]. There are two types of AD: early-onset AD that manifests in people under the age of 65 and the much more common late-onset AD that affects people over 65. The major risk factors for AD are advanced age, genetic predisposition, chronic diseases, head injuries, and other factors [2]. The histopathological characteristics in the AD brain include senile plaques composed of the extracellular accumulation of the amyloid *β* peptide and intraneuronal fibrillar aggregates of hyperphosphorylated tau proteins [3, 4].

The etiopathogenesis of AD remains unclear. One of the possible mechanisms of AD progression is related to neuroinflammation caused by matrix metalloproteinases (MMPs). MMPs are calcium-dependent zinc-containing endopeptidases that are involved in many physiological processes via cleavage of extracellular matrix components and basement membrane compounds. In the brain, MMPs play various roles involving neurogenesis, axonal growth, angiogenesis, tissue remodeling after injury, and inflammation [5]. To date, there are more than 25 MMPs described in humans. They are classified according to their abilities to cleave substrates or domain organization in collagenases (MMP-1, MMP-8, and MMP-13), gelatinases (MMP-2 and MMP-9), stromelysins (MMP-3, MMP-10, and MMP-11), matrilysins (MMP-7 and MMP-26), membrane-type (MT) MMPs (MMP-14, MMP-15, MMP-16, MMP-17, MMP-24, and MMP-25), and others (MMP-12, MMP-19, MMP-20, MMP-21, MMP-23, MMP-27, and MMP-28) [5].

The role of MMPs was studied in different neurodegenerative diseases such as multiple sclerosis (MS), Parkinson's disease (PD), amyotrophic lateral sclerosis (ALS), Huntington's disease (HD), and Alzheimer's disease [6]. In PD pathogenesis, MMP-3 has been involved in dopaminergic neurodegeneration, neuroinflammation, and barrier leakage [7]. Regarding MS, it was found that MMP-9 digests myelin basic protein, which causes demyelination and drives MS progression [8]. Furthermore, the MMP-9 degrade the endothelial basement membrane, which facilitates leukocyte extravasation and their migration into the brain [9]. The pathological role of MMP-9 was also reported in relation to amyotrophic lateral sclerosis. In the SOD1^G93A^ transgenic mouse model for ALS, genetic deletion of MMP-9 as well as its pharmacological inhibition has delayed muscle denervation [10]. In addition, some studies reported increased levels of MMP-9 in plasma and CSF of ALS patients considering MMP-9 as an early biomarker of the disease [11, 12]. Finally, MMP-3 and MMP-9 levels were increased in CSF from Huntington's disease patients and correlated with disease severity [13]. It was also found that MMP-10 cleaves huntingtin in the neurons to small N-terminal fragments thought to be toxic [14].

In our study, we have focused on MMP-2 and MMP-3 due to their involvement in the processes related to neurodegeneration. MMP-2 is a 72 kDa protein also known as gelatinase A. It is produced by most connective tissue cells including endothelial cells, osteoblasts, fibroblasts, and myoblasts cells. It is capable of hydrolyzing type IV collagen, which is the main component of the basement membrane followed by elastin, endothelin, fibroblast growth factor, plasminogen, TGF-*β*, and MMP-9 and MMP-13. The synthesis and secretion of MMP-2 can be stimulated by a variety of stimuli during various pathological processes, such as tumor invasion, atherosclerosis, and inflammation. In the brain, MMP-2 is crucial for neurite outgrowth and neuronal plasticity [15].

MMP-3, also known as stromelysin1, is a 54 kDa protein produced by various cells including the macrophages, stromal fibroblasts, endothelial cells, immune cells, and synovial cells. It cleaves an extensive range of extracellular matrix (ECM) molecules including collagen types 3, 4, 5, 9, 10, and 11, fibronectin, elastin, gelatins, laminins, and proteoglycans. In addition, it is involved in the proteolysis of various adhesion molecules like E-cadherin and L-selectin, growth factors like heparin-binding EGF-like growth factor, cytokines like TNF and IL-1*β*, and proforms of other MMPs like proMMP-1, proMMP-3, and proMMP-9. In the brain, MMP-3 is essential for neurite outgrowth, neuronal plasticity, and remyelination [16, 17].

Both MMP-2 and MMP-3 are assumed to be involved in AD pathogenesis. It was shown that AD patients have elevated levels of MMP-3 in the brain, especially in the microglia of white matter and senile plaques [18]. Increased MMP-3 levels in serum, plasma, and CSF of AD patients were observed as well [19–24]. In AD patients, there was an increase in MMP-2 expression in astrocytes surrounding amyloid plaque and a decrease in the MMP-2 plasma level compared to controls [25–27]. MMP-2 and MMP-3 were shown to cleave A*β* peptides to nontoxic fragments demonstrating a protective role in AD [28–30]. In addition, MMP-3 degrades tau protein preventing its aggregation [31]. On the other hand, increased MMP-2 and MMP-3 expression induced by toxic A*β*1-42 oligomers is related to the disruption of the blood-brain barrier (BBB) leading to neuroinflammation and AD progression [32–34].

It is known that the expression level may be influenced by functional polymorphic variants in the gene promoters. *MMP2* gene is located on chromosome 16q21 in the human genome, whereas *MMP3* gene is located on chromosome 11q22.3 [5]. *MMP2* rs243865 (-1306 C>T) and *MMP3* rs3025058 (-1171 5A>6A) are two common single nucleotide polymorphisms in the promoter region, which are associated with modified MMP expression levels. The role of *MMP2* and *MMP3* promoter gene polymorphisms in predisposition to AD development has been analysed in only few studies with controversial results. An association of *MMP3* -1171 5A allele and 5A/5A genotype with the risk of AD in *APOEε*4-positive patients was reported in two studies [35, 36]. Conversely, the association of *MMP3* -1171 6A allele with risk of AD was also found [37]. In other studies, no association of *MMP3* -1171 5A>6A polymorphism with AD susceptibility and clinical findings was observed [38–40].

Therefore, the objective of our study was to evaluate the association of *MMP2* rs243865 (-1306 C>T) and *MMP3* rs3025058 (-1171 5A>6A) polymorphisms with AD susceptibility and their influence on clinical findings in patients from Slovakia.

## 2. Materials and Methods

### 2.1. Study Groups

The investigated patient group included 171 unrelated individuals (53 men and 118 women, mean age: 79.68 ± 6.03 years) meeting criteria for late-onset Alzheimer's disease according to the ICD-10 classification [41]. AD patients were recruited at random via several psychiatric clinics throughout Slovakia. The average age at disease onset was 77.56 ± 6.39 years. The reference cohort in our case-control study comprised 308 unrelated volunteers (111 men and 197 women with a mean age of 76.23 ± 8.13 years). Montreal Cognitive Assessment (MoCA) was selected as the screening test for cognitive impairment in this study [42]. The cut-off score of 26 from 30 has been considered for normal cognition. Determination of the *APOEε*4 allele as a known genetic risk factor for AD was performed in both study groups and implemented as a stratification factor in further analyses. Detailed parameters of the study groups are summarized in Table 1.

All control individuals were without any personal or family history of AD, and they were randomly recruited from a larger population sample. All AD patients and controls were Caucasians of Slovak descent. Written informed consent for enrolling in the study and for personal data management was obtained from all AD patients or their legally authorized representatives as well as from the control subjects. All the investigations were carried out in accordance with the International Ethical Guidelines and the Declaration of Helsinki. The study was approved by the Independent Ethical Committee of the University Hospital Bratislava and the Faculty of Medicine, Comenius University in Bratislava.

### 2.2. Genotyping

Both patient and control DNAs were isolated from EDTA-treated whole blood by a modified salting out procedure [43]. Genotyping of *APOEε*4 allele was performed by the determination of rs429358 (C>T) and rs7412 (T>C) polymorphisms in the fourth exon using direct sequencing as described previously [44].

The *MMP2* rs243865 (-1306 C or T allele) was investigated by PCR followed by restriction fragment length polymorphism analysis (RFLP). Primer sequences, PCR conditions, and *XspI* (Thermo Fisher Scientific, U.S.A.) enzyme cleavage were used as reported by Benesova et al. [45]. A 188 bp product was amplified by PCR reaction. After digestion, either an intact 188 bp PCR fragment (allele C) or two fragments of 162 bp and 26 bp (allele T) were produced.

The *MMP3* rs3025058 (-1171 5A or 6A) was genotyped by PCR-RFLP as described by Dragovic et al. [46]. A 120 bp PCR product flanking the polymorphic site was amplified and afterwards digested with the restrictase *PdmI* (Thermo Fisher Scientific, U.S.A.). After digestion, either an intact 120 bp PCR fragment bearing allele with 6 adenines (6A) or two fragments of 97 bp and 23 bp consisting of 5 adenine (5A) allele were produced.

### 2.3. Statistical Analysis

Allele and genotype frequencies were determined by direct counting. Genotypes were tested for their fit to Hardy-Weinberg equilibrium using the chi-squared goodness-of-fit test. Statistical significance of differences in allele and genotype frequencies between AD patients and control group was evaluated by the Pearson chi-squared test using the InStat statistical software (GraphPad Software, Inc., San Diego, USA). The *p* values, odds ratios (OR), and 95% confidence intervals (95% CI) were calculated in codominant, dominant, recessive, and overdominant inheritance models. The multivariate logistic regression analysis adjusted for sex, age, and *APOEε*4 carriage status as possible modifying factors was performed by the SNPstats web software available at https://snapstat.net/snpstats/. The correlation between *MMP2* and *MMP3* gene promoter polymorphisms and clinical variables as age at onset and MoCA score was evaluated by the Student *t*-test with Welch correction. The *p* value of <0.05 was considered statistically significant.

## 3. Results

### 3.1. Characteristics of the Study Groups

The demographic and clinical characteristics of the study groups are shown in Table 1. 171 AD patients and 308 unrelated controls were included in the study. There was no statistically significant difference between the AD group and controls in relation to gender (*p* = 0.31), with females having higher prevalence in both AD patients (69.01%) and controls (63.96%). The mean age at examination was significantly higher in the AD group than in controls (79.68 versus 76.23 years; *p* < 0.0001), while an opposite trend was observed for the MoCA score having a lower value in AD patients (14.54 versus 27.52; *p* < 0.0001). The significantly higher prevalence of *APOEε*4 risk allele was found in the AD group compared to controls (39.18% vs 19.16%, *p* < 0.0001). The mean age at disease onset was 77.56 ± 6.39 years.

### 3.2. Genotyping of *MMP2* rs243865 and *MMP3* rs3025058 Polymorphisms in Promoter Region

Allele and genotype frequencies of *MMP2* rs243865 (-1306 C>T) and *MMP3* rs3025058 (-1171 5A>6A) observed in AD patients and control group are shown in Tables 2 and 3. Genotype frequencies of both polymorphisms fit the Hardy-Weinberg equilibrium in AD patients (*p* = 0.05 and *χ*^2^ = 4.13 for *MMP2*; *p* = 0.59 and *χ*^2^ = 0.29 for *MMP3*) as well as in controls (*p* = 0.56 and *χ*^2^ = 0.33 for *MMP2*; *p* = 0.17 and *χ*^2^ = 1.93 for *MMP3*). Genotyping of the SNP variants at *MMP2* -1306 C>T and at *MMP3* -1171 5A>6A revealed no statistically significant differences in either allele (*p* = 0.95, OR = 1.01 for *MMP2*; *p* = 0.68, OR = 1.07 for *MMP3*) or genotype (*p* > 0.05, OR = 0.82-1.37 for *MMP2*; *p* > 0.05, OR = 0.93-1.16 for *MMP3*) frequencies between the two studied groups. Multivariate analysis of association between the two SNPs and AD risk adjusted for age, sex, and *APOEε*4 positivity as potential confounding variables revealed no changes in comparison with the univariate analysis (*p* > 0.05, OR = 0.85-1.32 for *MMP2*, Table 2; *p* > 0.05, OR = 0.98-1.06 for *MMP3*, Table 3). Stratification of study groups according to their *APOEε*4 carriage status was also performed. Analyses in *APOEε*4-positive and *APOEε*4-negative groups revealed no statistically significant differences in the distribution of *MMP2* -1306 C>T and *MMP3* -1171 5A>6A genotypes between AD patients and control group (data not shown).

### 3.3. Association of *MMP2* rs243865 and *MMP3* rs3025058 Genotypes with Clinical Features in AD Patients

The association between *MMP2* rs243865 (-1306 C>T) and *MMP3* rs3025058 (-1171 5A>6A) genotypes and clinical features as age at disease onset and MoCA score was investigated. Correlation of clinical findings with *MMP2* -1306 C>T genotypes revealed that CC carriers in the dominant model had later age at disease onset when compared to T allele carriers (CC vs. CT+TT: 78.44 ± 6.28 vs. 76.36 ± 6.39, *p* = 0.036, Table 4). This association remained significant even after adjustment for sex and *APOEε*4 positivity (*p* = 0.024). Moreover, CT carriers in the overdominant model tended to have earlier disease onset as compared to other *MMP2* genotype carriers in adjusted models (CT vs. CC+TT: 76.23 ± 5.81 vs. 78.21 ± 6.58, *p* = 0.034, Table 4).

The correlation of investigated clinical findings with *MMP3* -1171 5A>6A genotypes is shown in Table 5. Statistical analysis revealed that 5A/6A carriers in the overdominant model seemed to have younger age at disease onset when compared to other *MMP3* genotype carriers (5A/6A vs. 5A/5A+6A/6A: 76.61 ± 5.88 vs. 78.57 ± 6.79, *p* = 0.045). After adjustment for sex and *APOEε*4 positivity, the significant association of 5A/6A carriers in the overdominant model with an earlier disease onset was also found (*p* = 0.048). On the other hand, the correlation of the MoCA score with both *MMP2* rs243865 and *MMP3* rs3025058 genotypes did not reveal any significant differences (*p* > 0.05, Tables 4 and 5).

## 4. Discussion

MMPs are zinc-containing endopeptidases that are suggested to be associated with the pathogenesis of many neurodegenerative diseases due to their involvement in microglial activation, T-leukocyte infiltration, and blood-brain barrier dysfunction [6]. In AD patients, both beneficial and detrimental effects of MMPs have been suggested. It has been reported that MMPs could degrade amyloid *β* and play important roles in the extracellular A*β* catabolism and clearance [28–30, 47]. On the other hand, MMPs could contribute to AD pathogenesis by disruption of the blood-brain barrier, cell apoptosis, and initiation of inflammation [33, 34].

The object of our study was MMP-2 and MMP-3 as candidate molecules related to AD pathology. In AD patients, an increase in MMP-2 and MMP-3 expression in the astrocytes surrounding amyloid plaques was reported [26, 48]. Moreover, increased MMP-3 levels in serum, plasma, and CSF in AD patients were also found [20–23]. Conversely, the decrease in MMP-2 plasma level in AD patients compared to controls was reported [26]. A negative correlation between MMP-3 plasma levels and the MMSE score was found [21].

As gene polymorphisms can modify gene expression and function, the aim of the study was to analyse the association of *MMP2* rs243865 and *MMP3* rs3025058 polymorphism with AD susceptibility and clinical findings in the Slovak Caucasian population. The *MMP2* rs243865 at position -1306 (C>T) and *MMP3* rs3025058 at position -1171 (5A>6A) in the promoter region have been associated with changes in MMP expression levels. The C to T transition at position -1306 prevents Sp1 binding to gene promoter leading to lower MMP-2 expression [49, 50]. Therefore, TT carriers have lower promoter activity and lower MMP-2 enzyme activity compared with CC carriers [51]. Regarding *MMP3* -1171 5A/6A polymorphism, its 5A allele has been reported to have greater transcriptional activity than the 6A allele [49].

To the best of our knowledge, genetic predisposition of *MMP2* rs243865 (-1306 C>T) and *MMP3* rs3025058 (-1171 5A>6A) to AD development has been analysed in only few studies. Our results showed no genetic association between *MMP2* rs243865 and *MMP3* rs3025058 polymorphism and AD susceptibility as reported by others [38–40]. On the other hand, studies in Finns and Italians reported a significantly higher occurrence of *MMP3* -1171 5A allele and 5A/5A genotype in *APOEε*4-positive AD patients [35, 36]. Moreover, Helbecque et al. [52] found association of *MMP3* -1171 6A/6A genotype with increased risk of dementia in *APOEε*4-negative AD patients from France. Finally, Baig et al. [37] reported association of *MMP3* -1171 6A allele with risk of AD in the UK. The discrepancies in genetic differences within the AD populations could reflect differences in various European regions or may be caused by differences in sample sizes, study design, and statistical methods.

In this study, the analysis of association of *MMP2* rs243865 (-1306 C>T) and *MMP3* rs3025058 (-1171 5A>6A) genotypes with clinical features such as age at onset and MoCA score was also performed. We found a significant association of *MMP2* -1306 CC genotype in the dominant model with later age at disease onset in crude analysis and adjusted models. Furthermore, we observed that *MMP3* -1171 5A/6A carriers in the overdominant model tended to have lower age at disease onset when compared to other *MMP3* genotype carriers. As *MMP2* -1306 C allele is associated with higher promoter activity, it can be hypothesized that CC genotype has protective effect on AD development. Furthermore, the -1171 5A allele as a high MMP-3 producer has been associated with the pathogenesis of various diseases like acute myocardial infarction [53], breast cancer [54, 55], head and neck squamous cell carcinoma [56], and lung cancer [57].

The impact of *MMP2* rs243865 and *MMP3* rs3025058 on clinical features, including age at onset and MoCA score, has not yet been reported. Reitz et al. [39] performed an analysis of *MMP3* polymorphism with clinical findings such as cognitive MMSE performance over time, hippocampal volume, or severity of periventricular and subcortical white matter lessions. They did not find any correlation of *MMP3* genotypes or haplotypes with the above-mentioned clinical features [39]. Another study by Reitz et al. [40] investigated the association of *MMP3* -1171 5A>6A, 2092A>G, 9775T>A, and 6658T>C SNPs and their haplotypes with plasma A*β*1–40 and A*β*1–42 levels in AD patients [40]. There was no association between *MMP3* -1171 5A>6A or 6658T>C and A*β*1–40 or A*β*1–42 levels in crude or adjusted models. However, haplotype analysis showed that haplotype 2 (6A-G-T-T) was linked with significantly higher levels of plasma A*β*1–42 as compared with haplotype 1 (5A-A-T-T) (*p* = 0.0002) [40].

The role of MMP-2 and MMP-3 expression levels in AD pathology is not yet well defined. It seems that decreased MMP-2 levels in AD patients correlate with impaired degradation of A*β* peptides [58]. Conversely, A*β*-induced expression of MMP-2 and MMP-3 may contribute to the breakdown of BBB and induction of neuroinflammation [32–34]. A recent study described that A*β*-induced MMP-3 may contribute to NGF degradation leading to cholinergic atrophy and cognitive deficits in AD males [59]. Thus, it is unclear whether changes in MMP levels contribute to AD progression or might have beneficial effects on AD patients.

As MMPs seem to be involved in AD pathogenesis, their utility as therapeutic targets has been also investigated. One possibility relies on promoting MMP activities resulting in A*β* degradation. In the APP mouse model, a novel rhamnoside derivative PL402 was reported to promote A*β* cleavage via upregulation of MMP-3/MMP-9 [60]. However, this therapeutic approach should be taken with caution as it cannot be excluded that proteolytic degradation of amyloid plaques could release toxic A*β* products and other neurotoxins. On the other hand, GM6001 and another MMP inhibitor, minocycline, were reported to efficiently reduce upregulated MMP-2 and MMP-9 and prevent inflammation and oxidative stress associated with cerebral amyloid angiopathy in AD mice [61, 62].

We intend to further investigate other MMPs, such as MMP-9, which will also help to understand their role in AD etiology. Similarly to MMP-2 and MMP-3, MMP-9 could also act in A*β* degradation thus preventing deposition of A*β* [30]. MMP-9 is also involved in BBB disruption and induction of neuroinflammation [63]. *MMP9* rs3918242 at position -1562C/T is another candidate polymorphism for AD susceptibility. It was found that this C to T substitution prevents the binding of a nuclear transcription repressor protein to the *MMP9* gene promoter thus increasing its transcription [64]. A protective effect of the *MMP9* -1562 T allele having greater promoter activity in *APOEε*4-negative AD patients was reported [65]. Thus, *MMP* gene promoter variants seem to be promising biomarkers whose role in the pathogenesis of AD warrants further investigations.

## 5. Conclusion

Among various MMPs, MMP-2 and MMP-3 belong to candidate molecules involved in AD pathogenesis. This is the first study investigating the impact of *MMP2* rs243865 and *MMP3* rs3025058 promoter polymorphisms on clinical features, including age at onset and MoCA score in AD patients. While no genetic association of *MMP2* rs243865 and *MMP3* rs3025058 with the risk of AD was found, our results suggest that *MMP2* rs243865 CC genotype and *MMP3* rs3025058 5A/6A genotype may have influence on the age of AD onset. In conclusion, *MMP2* rs243865 and *MMP3* rs3025058 polymorphisms may contribute to modification of certain clinical parameters in AD patients.

## Figures and Tables

**Table 1:** Demographic and clinical characteristics of AD patients and controls.

Parameter	AD subjects (*n* = 171)	Controls (*n* = 308)	*p* value
Female/male ratio	118/53	197/111	0.31
Age at examination (y); mean ± SD	79.68 ± 6.03	76.23 ± 8.13	<0.0001
Age at onset (y); mean ± SD	77.56 ± 6.39	—	—
MoCA score, mean ± SD	14.54 ± 5.80	27.52 ± 1.44	<0.0001
*APOEε*4 positivity (yes/no)	67/104	59/249	<0.0001

Abbreviations: *n*: number; SD: standard deviation; MoCA: Montreal Cognitive Assessment; y: years. Differences in age and MoCA score between the two groups were examined by Welch's corrected *t*-test. Differences in sex were assessed using the Pearson chi-squared test. *p* < 0.05 is considered statistically significant.

**Table 2:** Allele and genotype frequencies of *MMP2* polymorphism rs243865 (-1306 C/T) in AD patients and controls.

SNP/model	Allele/genotype	AD subjects (*n* = 171)	Controls (*n* = 308)	Univariate analysis		Multivariate analysis	
				*p*	OR (95% CI)	*p*	OR (95% CI)
rs243865	C	252 (73.68%)	455 (73.86%)			—	—
	T	90 (26.32%)	161 (26.14%)	0.95	1.01 (0.75-1.36)	—	—

Codominant	CC	98 (57.31%)	170 (55.19%)		1.00		1.00
	CT	56 (32.75%)	115 (37.34%)	0.46	0.84 (0.56-1.27)	0.62	0.88 (0.57-1.34)
	TT	17 (9.94%)	23 (7.47%)		1.28 (0.65-2.52)		1.25 (0.61-2.57)

Dominant	CC	98 (57.31%)	170 (55.19%)		1.00		1.00
	CT+TT	73 (42.69%)	138 (44.81%)	0.65	0.92 (0.63-1.34)	0.76	0.94 (0.63-1.40)

Recessive	CC+CT	154 (90.06%)	285 (92.53%)		1.00		1.00
	TT	17 (9.94%)	23 (7.47%)	0.35	1.37 (0.71-2.64)	0.44	1.32 (0.65-2.65)

Overdominant	CC+TT	115 (67.25%)	193 (62.66%)				
	CT	56 (32.75%)	115 (37.34%)	0.31	0.82 (0.55-1.21)	0.44	0.85 (0.56-1.29)

Abbreviations: CI: confidence interval; *n*: number; OR: odds ratio. Allele and genotype frequencies are given as absolute numbers with percentages in parentheses. Univariate analysis is based on the Pearson chi-squared test. Multivariate analysis is adjusted for sex, age, and *APOEε*4 positivity. *p* < 0.05 is considered statistically significant.

**Table 3:** Allele and genotype frequencies of *MMP3* polymorphism rs3025058 (-1171 5A/6A) in AD patients and controls.

SNP/model	Allele/genotype	AD subjects (*n* = 171)	Controls (*n* = 308)	Univariate analysis		Multivariate analysis	
				*p*	OR (95% CI)	*p*	OR (95% CI)
rs3025058	6A	171 (50.00%)	318 (51.62%)			—	—
	5A	171 (50.00%)	298 (48.38%)	0.68	1.07 (0.82-1.39)	—	—

Codominant	6A/6A	41 (23.98%)	76 (24.67%)		1.00		1.00
	5A/6A	89 (52.04%)	166 (53.90%)	0.82	0.99 (0.63-1.57)	0.98	1.00 (0.62-1.63)
	5A/5A	41 (23.98%)	66 (21.43%)		1.15 (0.67-1.98)		1.06 (0.59-1.88)

Dominant	6A/6A	41 (23.98%)	76 (24.67%)		1.00		1.00
	5A/6A+5A/5A	130 (76.02%)	232 (75.33%)	0.86	1.04 (0.67-1.61)	0.94	1.02 (0.64-1.62)

Recessive	6A/6A+5A/6A	130 (76.02%)	242 (78.57%)		1.00		1.00
	5A/5A	41 (23.98%)	66 (21.43%)	0.52	1.16 (0.74-1.80)	0.82	1.05 (0.66-1.68)

Overdominant	5A/5A+6A/6A	82 (47.96%)	142 (46.10%)				
	5A/6A	89 (52.04%)	166 (53.90%)	0.70	0.93 (0.64-1.35)	0.90	0.98 (0.66-1.45)

Abbreviations: CI: confidence interval; *n*: number; OR: odds ratio. Allele and genotype frequencies are given as absolute numbers with percentages in parentheses. Univariate analysis is based on the Pearson chi-squared test. Multivariate analysis is adjusted for sex, age, and *APOEε*4 positivity. *p* < 0.05 is considered statistically significant.

**Table 4:** Analysis of association between *MMP2* rs243865 (-1306 C/T) genotypes and clinical findings.

Parameter	CC (*n* = 98)	CT (*n* = 56)	TT (*n* = 17)	*p*/*p*^∗^ CM	*p*/*p*^∗^ DM	*p*/*p*^∗^ RM	*p*/*p*^∗^ OM
Age at onset, mean ± SD (y)	78.44 ± 6.28	76.23 ± 5.81	76.81 ± 8.32	0.11/0.07	0.036/0.024	0.62/0.68	0.058/0.034
MoCA score, mean ± SD	15.20 ± 6.01	13.56 ± 5.68	14.54 ± 4.03	0.32/0.29	0.16/0.13	1.00/0.92	0.14/0.13

Abbreviations: CM: codominant model; DM: dominant model; RM: recessive model; OM: overdominant; SD: standard deviation; MoCA: Montreal Cognitive Assessment; *n*: number; y: years. *p* values were calculated using Welch's corrected *t*-test. ^∗^*p* values adjusted for sex and *APOEε*4 positivity. *p* < 0.05 is considered statistically significant.

**Table 5:** Analysis of association between *MMP3* rs3025058 (-1171 5A/6A) genotypes and clinical findings.

Parameter	5A/5A (*n* = 41)	5A/6A (*n* = 89)	6A/6A (*n* = 41)	*p*/*p*^∗^ CM	*p*/*p*^∗^ DM	*p*/*p*^∗^ RM	*p*/*p*^∗^ OM
Age at onset, mean ± SD (y)	79.02 ± 5.51	76.61 ± 5.88	78.12 ± 7.91	0.11/0.11	0.09/0.09	0.52/0.53	0.045/0.048
MoCA score, mean ± SD	14.65 ± 4.55	14.25 ± 6.10	14.97 ± 6.56	0.84/0.88	0.89/0.85	0.63/0.71	0.59/0.63

Abbreviations: CM: codominant model; DM: dominant model; RM: recessive model; OM: overdominant; SD: standard deviation; MoCA: Montreal Cognitive Assessment; *n*: number; y: years. *p* values were calculated using Welch's corrected *t*-test. ^∗^*p* values adjusted for sex and *APOEε*4 positivity. *p* < 0.05 is considered statistically significant.

## Data Availability

The data used to support the findings of this study are included within the article.

## Abbreviations

AD: Alzheimer's disease
ALS: Amyotrophic lateral sclerosis
APOE: Apolipoprotein E
APP: Amyloid *β* precursor protein
A*β*: Amyloid *β*
BBB: Blood-brain barrier
CSF: Cerebrospinal fluid
ECM: Extracellular matrix
HD: Huntington's disease
MMP: Matrix metalloproteinase
MMSE: Minimental state examination
MoCA: Montreal Cognitive Assessment
MS: Multiple sclerosis
NGF: Nerve growth factor
PD: Parkinson's disease
RFLP: Restriction fragment length polymorphism analysis
TGF-*β*: Transforming growth factor-*β*
TNF: Tumor necrosis factor.
